# Supplementary material for: Urine proteomic analysis of the rat e-cigarette model
Source: PeerJ. 2023 Sep 22;11:e16041. doi: 10.7717/peerj.16041 (PMC10519197; doi:10.7717/peerj.16041)
Supplement: Supplemental Information 1 [file peerj-11-16041-s001.pdf]

Table S1. Differential proteins identified in the D3 test group before and after self-control in 6 rats

UniProt accession	Human ortholog	Protein name	Fold change(vs D0)					
			Rat 1	Rat 2	Rat 3	Rat 4	Rat 5	Rat 6
P08721	P10451	Osteopontin	3.29	3.60	2.93	0.65	2.07	0.55
A0A0G2JYF9	O15232	Matrilin 3	4.00	2.11	2.24	2.93	2.51	0.44
P07151	P61769	Beta-2-microglobulin	1.91	1.73	2.23	0.62	2.35	-
Q6IRS6	Q9UGM5	Fetuin-B	0.47	0.40	0.47	0.32	0.31	-
Q9QYU9	-	Odorant-binding protein 1F	0.53	0.46	0.41	0.65	-	0.26
Q64724	-	C-CAM4	0.59	0.67	0.53	0.40	-	0.22
B3EY84	Q9NY56	Lipocalin 13, odorant-binding protein 2A	0.59	0.59	0.39	0.63	-	0.22
P22006	-	Seminal vesicle secretory protein 2	0.37	3.97	9.74	0.12	-	0.09
F1LP05	-	ATP synthase subunit alpha	0.38	0.45	0.31	-	2.56	5.19
Q07936	P07355	Annexin A2	0.60	4.19	-	1.59	1.82	2.39
P05545	-	Serine protease inhibitor A3K	0.62	0.60	-	0.64	0.56	0.46
D4AE68	-	Guanine nucleotide-binding protein G(q) subunit alpha	-	0.57	0.28	2.32	2.84	4.03
G3V803	P19022	Cadherin-2, Neural cadherin	-	0.65	0.56	0.59	0.57	0.32
P46413	P48637	Glutathione synthetase	-	0.42	0.50	0.42	0.52	0.45
Q5I0D7	P12955	Xaa-Pro dipeptidase	-	0.39	0.59	0.44	0.65	0.45
