# Supplementary material for: Urine proteomic analysis of the rat e-cigarette model
Source: PeerJ. 2023 Sep 22;11:e16041. doi: 10.7717/peerj.16041 (PMC10519197; doi:10.7717/peerj.16041)
Supplement: Supplemental Information 2 [file peerj-11-16041-s002.pdf]

Table S2. Differential proteins identified in the D12 test group before and after self-control in 6 rats

**UniProt accession**	**Human ortholog**	**Protein name**	**Fold change(vs D0)**					
			**Rat 1**	**Rat 2**	**Rat 3**	**Rat 4**	**Rat 5**	**Rat 6**
D4AE68	-	Guanine nucleotide-binding protein G(q) subunit alpha	2.89	1.80	0.50	3.07	1.57	4.83
Q6IRS6	Q9UGM5	Fetuin-B	0.42	0.57	0.62	0.53	0.53	0.33
M0RDH1	-	Odorant-binding protein 2B	2.49	2.20	2.14	2.90	2.17	0.46
G3V803	P19022	Cadherin-2, Neural cadherin	0.47	0.54	0.42	0.57	0.58	0.22
D4A9V5	-	Lysyl oxidase homolog	0.54	0.40	0.67	0.47	0.41	0.20
P07151	P61769	Beta-2-microglobulin	2.75	2.07	2.84	0.57	2.08	2.19
A0A0G2K230	Q14574	Desmocollin 3	1.96	2.73	4.04	2.41	3.02	-
P51635	P14550	Aldo-keto reductase family 1 member A1	2.29	1.62	1.96	2.87	3.40	-
P14668	P08758	Annexin A5	1.56	1.84	2.92	2.96	5.95	-
G3V8V1	-	Granulin, isoform CRA_c	0.54	0.58	0.52	-	0.22	0.32
A0A0G2K6Z6	-	Tartrate-resistant acid phosphatase type 5	0.38	0.39	0.49	-	0.29	0.27
P20761	-	Ig gamma-2B chain C region	0.33	1.79	1.78	-	1.69	2.55
Q68FP1	P06396	Gelsolin	2.20	1.82	2.10	-	2.51	2.38
F1LPR6	-	Immunoglobulin heavy constant epsilon	2.67	2.85	-	2.02	2.94	3.33
F1M5L5	-	Ig-like domain-containing protein	3.89	1.75	-	2.78	1.97	0.53
P12020	P54107	Cysteine-rich secretory protein 1	2.38	2.41	-	0.13	4.77	4.95
F1M8K0	-	Dystroglycan 1	0.50	0.47	-	0.23	0.62	0.47
Q6VPP3	-	Chloride channel accessory 4	0.62	0.30	-	0.39	1.61	0.65
G3V8T4	-	DNA damage-binding protein 1	2.51	1.69	-	4.95	1.99	2.72
Q99041	P49221	Protein-glutamine gamma-glutamyltransferase 4	-	0.05	0.04	0.06	0.24	16.03
P0DMW0	P0DMV8	Heat shock 70 kDa protein 1A	-	2.75	2.41	0.33	5.65	3.30
