# Supplementary material for: Urine proteomic analysis of the rat e-cigarette model
Source: PeerJ. 2023 Sep 22;11:e16041. doi: 10.7717/peerj.16041 (PMC10519197; doi:10.7717/peerj.16041)
Supplement: Supplemental Information 3 [file peerj-11-16041-s003.pdf]

Table S3. Differential proteins identified in the D15 test group before and after self-control in 6 rats

**UniProt accession**	**Human ortholog**	**Protein name**	**Fold change(vs D0)**					
			**Rat1**	**Rat2**	**Rat3**	**Rat4**	**Rat5**	**Rat6**
Q6IRS6	Q9UGM5	Fetuin-B	0.65	0.45	0.43	0.42	0.39	0.32
B3EY84	Q9NY56	Lipocalin 13, odorant-binding protein 2A	1.88	2.94	1.77	1.68	2.53	0.61
D3ZS19	-	Alpha-2-macroglobulin-like 1	0.63	0.57	0.60	1.69	2.03	4.55
P10960	P07602	Prosaposin	2.35	1.93	1.77	0.58	0.63	0.27
Q9JJI3	-	Alpha-2u globulin	10.57	1.68	11.56	2.54	4.85	20.87
P07151	P61769	Beta-2-microglobulin	6.87	2.43	2.81	1.74	4.07	2.70
D3ZE05	-	Cartilage intermediate layer protein 2	0.56	0.27	0.20	0.55	0.29	-
D4A183	-	Similar to Vanin-3	2.76	6.24	5.36	5.63	2.80	-
P27590	P07911	Uromodulin	3.73	4.67	4.08	4.81	3.75	-
Q8K4J7	-	Resistin	2.22	2.88	2.74	0.66	1.58	-
D4A133	-	H(+)-transporting two-sector ATPase	0.51	0.46	0.57	2.79	1.56	-
P80020	P51161	Gastrotropin	5.16	1.51	2.54	18.35	-	15.76
F1LZ11	-	Ig-like domain-containing protein	0.55	2.32	4.12	2.63	-	3.97
A0A0G2JWD0	-	Prominin 1	0.24	0.57	0.65	1.78	-	2.59
A0A0G2K2R5	-	EGF-containing fibulin extracellular matrix protein 2	0.42	0.59	0.42	0.33	-	1.63
F7F389	-	Complement component C9	1.59	2.28	1.51	2.29	-	2.31
P14668	P08758	Annexin A5	1.58	2.07	2.80	1.88	-	4.62
F1M9X2	-	Pancreatic secretory granule membrane major glycoprotein GP2	1.50	3.22	3.04	0.53	-	0.45
P07171	P05937	Calbindin	3.64	4.36	1.58	0.53	-	0.43
P46413	P48637	Glutathione synthetase	0.39	0.52	0.39	0.51	-	0.48
P42854	Q6UW15	Regenerating islet-derived protein 3-gamma	3.39	4.12	2.87	1.68	-	0.32
Q99041	P49221	Protein-glutamine gamma-glutamyltransferase 4	0.20	0.07	2.33	-	7.78	6.79
P55159	P27169	Serum paraoxonase/arylesterase 1	0.44	0.57	0.53	-	0.39	1.52
Q8R5M3	Q8TF66	Leucine-rich repeat-containing protein 15	0.38	0.32	0.45	-	0.66	3.21
F1LQQ8	-	Beta-glucuronidase	11.84	1.57	7.06	-	5.79	5.25
A0A0G2K896	-	Similar to RIKEN cDNA 1300017J02	0.55	0.57	-	1.58	1.86	10.58
M0R3V4	-	Myeloid-derived growth factor	3.83	2.86	-	2.94	3.30	2.28
B2RZ27	Q9H299	SH3 domain-binding glutamic acid-rich-like protein 3	0.63	0.51	-	0.57	0.47	0.45
A0A0G2JSV2	-	Carbonyl reductase	0.47	1.62	-	6.72	4.47	2.96
D4A9V5	-	Lysyl oxidase homolog	0.53	0.51	-	0.25	0.48	0.14
F1M6Q3	-	Collagen type IV alpha 2 chain	0.44	0.51	-	0.35	0.52	2.30

F7F5P4	-	Interleukin 18-binding protein	0.53	-	0.47	0.34	0.53	0.23
G3V803	P19022	Cadherin-2, Neural cadherin	0.54	-	0.50	0.45	0.51	0.27
Q9JJH9	-	Alpha-2u globulin	9.11	-	13.25	1.59	4.04	20.32
D4A144	-	Adhesion G protein-coupled receptor L1	-	0.57	0.43	0.34	0.53	0.34
Q63083	Q02818	Nucleobindin-1	-	1.91	1.98	0.51	0.61	0.33
P20761	-	Ig gamma-2B chain C region	-	3.56	2.80	1.90	2.08	3.41
