# Supplementary material for: Urine proteomic analysis of the rat e-cigarette model
Source: PeerJ. 2023 Sep 22;11:e16041. doi: 10.7717/peerj.16041 (PMC10519197; doi:10.7717/peerj.16041)
Supplement: Supplemental Information 4 [file peerj-11-16041-s004.pdf]

Table S4. Differential proteins identified in the D17 test group before and after self-control in 6 rats

UniProt accession	Human ortholog	Protein name	Fold change(vs D0)					
			Rat1	Rat2	Rat3	Rat4	Rat5	Rat6
Q99041	P49221	Protein-glutamine gamma-glutamyltransferase 4	3.27	0.02	0.65	0.07	0.15	2.02
Q9JJH9	-	Alpha-2u globulin	1.65	9.42	9.40	4.18	9.97	28.93
Q68FP1	P06396	Gelsolin	2.11	1.92	2.00	2.48	0.61	2.33
Q6MG70	-	Exo-alpha-sialidase	0.63	0.42	0.46	0.50	0.40	-
M0R9A3	-	PBPC1BS-like	0.34	0.62	0.56	0.38	3.11	-
M0RDH1	-	Odorant-binding protein 2B	2.68	2.52	2.71	3.26	8.20	-
A0A0G2K230	Q14574	Desmocollin 3	2.17	3.12	4.04	3.27	8.20	-
P27590	P07911	Uromodulin	4.77	4.02	2.65	4.66	13.14	-
B3EY84	Q9NY56	Lipocalin 13, Odorant-binding protein 2A	2.13	1.64	1.76	2.21	8.61	-
P08290	P07307	Asialoglycoprotein receptor 2	0.43	0.27	0.63	0.29	-	0.23
P10758	-	Lithostathine	2.85	8.60	6.04	3.68	-	5.29
P20761	-	Ig gamma-2B chain C region	0.31	3.85	2.40	2.59	-	2.95
G3V803	P19022	Cadherin-2, Neural cadherin	0.57	0.39	0.57	0.43	-	0.19
P0DMW0	P0DMV8	Heat shock 70 kDa protein 1A	0.67	3.06	2.22	0.33	-	4.77
A0A0G2K6T9	-	Protocadherin 1	0.32	0.29	0.38	0.34	-	0.13
A0A0G2K7W2	P43353	Aldehyde dehydrogenase	0.61	0.17	0.22	-	3.24	0.48
O55004	P34096	Ribonuclease 4	0.63	2.22	-	1.72	3.56	0.62
Q07936	P07355	Annexin A2	0.58	4.31	-	0.65	0.15	5.30
A0A140TAF0	-	Tropomyosin 3	3.53	15.99	-	11.38	40.92	3.48
P19132	P02794	Ferritin heavy chain	0.59	0.37	-	0.37	6.60	3.87
G3V6G1	-	Immunoglobulin joining chain	3.99	-	2.85	1.66	17.34	2.55
A0A0G2K405	P13861	cAMP-dependent protein kinase type II-alpha regulatory subunit	1.98	-	0.54	1.73	0.65	4.22
P12368	P13861	cAMP-dependent protein kinase type II-alpha regulatory subunit	1.98	-	0.54	1.73	0.65	4.22
P05545	-	Serine protease inhibitor A3K	0.65	-	0.53	0.53	0.58	0.38
P51635	P14550	Aldo-keto reductase family 1 member A1	2.13	-	1.51	1.74	0.22	4.03
P12346	P02787	Serotransferrin	0.60	-	0.66	0.60	0.11	4.34
Q00238	P05362	Intercellular adhesion molecule 1	-	0.65	0.63	0.58	2.18	0.42
Q9WUC4	O00244	Copper transport protein ATOX1	-	0.66	1.73	0.62	4.80	0.56

Q64724	-	C-CAM4	-	0.66	0.64	0.42	1.96	0.39
P14668	P08758	Annexin A5	-	1.64	2.94	1.59	0.18	6.35
