# Supplementary material for: Urine proteomic analysis of the rat e-cigarette model
Source: PeerJ. 2023 Sep 22;11:e16041. doi: 10.7717/peerj.16041 (PMC10519197; doi:10.7717/peerj.16041)
Supplement: Supplemental Information 5 [file peerj-11-16041-s005.pdf]

Table S5. Biological processes enriched by the differential proteins identified jointly before and after comparison in five or more treated rats

| Biological process | P-value | | | |
|---|---|---|---|---|
| | D3 | D12 | D15 | D17 |
| negative regulation of endopeptidase activity | 7.00E-03 | - | - | - |
| negative regulation of endothelial cell proliferation | 3.10E-02 | - | - | - |
| response to cadmium ion | 3.70E-02 | - | 8.70E-02 | - |
| antibacterial humoral response | 4.80E-02 | - | - | 9.40E-02 |
| antimicrobial humoral immune response mediated by antimicrobial peptide | 7.40E-02 | - | - | - |
| protein stabilization | - | 2.90E-05 | - | 3.30E-02 |
| lysosomal transport | - | 1.50E-04 | - | 2.80E-02 |
| protein refolding | - | 2.20E-04 | - | 3.40E-02 |
| positive regulation of endoribonuclease activity | - | 1.90E-03 | - | 3.00E-03 |
| cellular heat acclimation | - | 4.80E-03 | - | 7.50E-03 |
| positive regulation of nucleotide-binding oligomerization domain containing 2 signaling pathway | - | 5.80E-03 | - | 9.00E-03 |
| negative regulation of transcription from RNA polymerase II promoter in response to stress | - | 5.80E-03 | - | 9.00E-03 |
| positive regulation of microtubule nucleation | - | 8.60E-03 | - | 1.30E-02 |
| negative regulation of inclusion body assembly | - | 9.60E-03 | - | 1.50E-02 |
| positive regulation of tumor necrosis factor-mediated signaling pathway | - | 1.20E-02 | - | 1.90E-02 |
| amyloid fibril formation | - | 1.30E-02 | - | - |
| vesicle-mediated transport | - | 1.40E-02 | - | 3.20E-02 |
| negative regulation of mitochondrial outer membrane permeabilization involved in apoptotic signaling pathway | - | 1.40E-02 | - | 2.20E-02 |
| negative regulation of apoptotic process | - | 1.80E-02 | - | - |
| positive regulation of RNA splicing | - | 1.80E-02 | - | 2.80E-02 |
| chaperone-mediated protein complex assembly | - | 2.00E-02 | - | 3.10E-02 |
| negative regulation of endoplasmic reticulum stress-induced intrinsic apoptotic signaling pathway | - | 2.00E-02 | - | 3.10E-02 |
| response to ethanol | - | 2.00E-02 | - | 4.60E-02 |
| regulation of mitotic spindle assembly | - | 2.10E-02 | - | 3.20E-02 |
| regulation of protein ubiquitination | - | 2.10E-02 | - | 3.20E-02 |
| cellular response to unfolded protein | - | 2.20E-02 | - | 5.40E-04 |
| response to unfolded protein | - | 3.20E-02 | - | 1.20E-03 |
| chaperone mediated protein folding requiring cofactor | - | 3.20E-02 | - | 5.00E-02 |
| positive regulation of erythrocyte differentiation | - | 3.30E-02 | - | 5.10E-02 |
| negative regulation of extrinsic apoptotic signaling pathway in absence of ligand | - | 3.50E-02 | - | 5.40E-02 |
| mRNA catabolic process | - | 3.60E-02 | - | 5.50E-02 |
| positive regulation of T cell-mediated cytotoxicity | - | 3.70E-02 | - | - |
| binding of sperm to zona pellucida | - | 4.60E-02 | - | - |

| | | | | |
|---|---|---|---|---|
| ATP metabolic process | - | 4.70E-02 | - | 7.20E-02 |
| negative regulation of protein ubiquitination | - | 5.40E-02 | - | 8.30E-02 |
| cellular response to heat | - | 5.50E-02 | - | 8.50E-02 |
| positive regulation of interleukin-8 production | - | 6.20E-02 | - | 9.40E-02 |
| phagocytosis, engulfment | - | 6.60E-02 | - | - |
| positive regulation of proteasomal ubiquitin-dependent protein catabolic process | - | 7.20E-02 | - | - |
| negative regulation of transforming growth factor beta receptor signaling pathway | - | 7.40E-02 | - | - |
| response to heat | - | 8.30E-02 | - | - |
| heterophilic cell–cell adhesion via plasma membrane cell adhesion molecules | - | - | 2.90E-03 | 6.40E-02 |
| innate immune response | - | - | 5.60E-03 | - |
| complement activation, classical pathway | - | - | 6.40E-03 | - |
| metanephric distal convoluted tubule development | - | - | 7.30E-03 | - |
| vitamin K metabolic process | - | - | 9.10E-03 | - |
| micturition | - | - | 1.10E-02 | - |
| defence response to gram-negative bacterium | - | - | 1.30E-02 | - |
| glucocorticoid metabolic process | - | - | 1.60E-02 | - |
| neutrophil migration | - | - | 3.10E-02 | - |
| retina layer formation | - | - | 4.60E-02 | - |
| positive regulation of MAPK cascade | - | - | 5.10E-02 | - |
| positive regulation of reactive oxygen species metabolic process | - | - | 6.50E-02 | - |
| iron ion transport | - | - | 6.90E-02 | 5.70E-02 |
| cochlea development | - | - | 7.20E-02 | - |
| positive regulation of B cell activation | - | - | 8.40E-02 | - |
| cellular response to organic substance | - | - | 8.60E-02 | - |
| phagocytosis, recognition | - | - | 9.10E-02 | - |
| response to lipopolysaccharide | - | - | 9.10E-02 | - |
| cellular aldehyde metabolic process | - | - | - | 4.50E-05 |
| aldehyde catabolic process | - | - | - | 4.50E-03 |
| lipid metabolic process | - | - | - | 1.90E-02 |
| homophilic cell adhesion via plasma membrane adhesion molecules | - | - | - | 2.20E-02 |
| positive regulation of NF-kappaB transcription factor activity | - | - | - | 2.40E-02 |
| response to thyroid hormone | - | - | - | 2.80E-02 |
| cell–cell adhesion | - | - | - | 3.00E-02 |
| negative regulation of cell proliferation | - | - | - | 3.00E-02 |
| glomerular filtration | - | - | - | 3.10E-02 |
| leukocyte cell–cell adhesion | - | - | - | 4.50E-02 |
| retina homeostasis | - | - | - | 5.30E-02 |
| positive regulation of gene expression | - | - | - | 6.00E-02 |
| calcium ion homeostasis | - | - | - | 6.70E-02 |
| response to hypoxia | - | - | - | 6.80E-02 |

| | | | | |
|---|---|---|---|---|
| cellular iron ion homeostasis | - | - | - | 7.50E-02 |
| response to organic cyclic compound | - | - | - | 8.40E-02 |
